# Babies

**Published:** 1869-07

**Authors:** 


### BABIES.

Who would be without a baby?—Not we—they are necessary to the enjoyment of every household, and we pity the family who are trying to keep house without one. Babies are jolly—indeed, we might add, were we given to vulgar expressions, that babies are bully! But that being inelegant, we content ourself by merely asserting that babies are splendid. Now, we might just here remark that we've got a baby—therefore are we blest, and that accounts for us opening the subject. Our baby is not as young as he was some eighteen months ago, but he is sufficiently lively for all practical purposes. Indeed, our baby might be called an acrobatic baby, for the *way* he throws somersaults and performs other gymnastic feats is truly remarkable in one of his stature. Our baby is useful, too, as well as playful. We have long since abandoned winding our alarm clock, for baby fulfills its office, and more too, for he never runs down, but may be discovered at *any* hour of the night in his infantile acrobatic *role*. This is a wonderful convenience, for we are certain to be awake at any given hour of the night. Baby is very loving in his disposition, too—he twines his little fat legs about our neck and pokes his curly little head into our face until we forget the lateness of the hour or our disposition to sleep, and laugh in spite of ourself. Baby has learned to play "horse"—he chooses the early morning for his equestrian feats—usually about three o'clock,—sits astride our neck and uses our auburn locks for a bridle. Dreaming that we are about being suffocated in some chemist's laboratory, and that a cloth of liquid amonia is being held to our nose for resuscitating purposes, we awake in a state of alarm, only to find baby in his accustomed seat digging his heels into our ribs and singing ge-up! ge-up! Baby is teething—cutting his "eye teeth," in fact—and stoutly insists upon using our proboscis or our left ear upon which to impress his canines. We cannot object to this, for if we do, baby cries, and his mamma thinks it is real cruel to allow babies to cry just for the want of something to chew, so we allow him to choose his own means of enjoyment—and that accounts for our red nose, although Amalichi John asserts that it is from the too free use of Bass' ale notwithstanding we protest "it's not what ails our nose". But this is a digression—we return to our subject—Babies.

Babies are not as numerous as they used to be—when we were young, mothers used to have six or eight babies—but now, alas, babies *have* their mothers. In other words *one* baby is as much as a mother can manage now-a-days, and then, she has to call in the assistance of somebody's else mother to care for that one.—Formerly a mother used to raise from eight to twelve children and do her own house-work too. *Now* she has *no* children and hires a half-dozen grown up ones to do her housekeeping for her, which leads us to remark that things have changed!

Some say babies are expensive. That depends upon whether you have a *doll* baby or a real, live *healthy* one. *Our* baby doesn't wear many clothes to speak of—we don't keep him for show, but for genuine comfort. Some would call him a dirty baby—*we* don't think so, for we can pick him up from his play with Jocko, and the chickens in the back yard—brush his face with our long coat tail to see whether it is he or not, and kiss his little pouting lips with real pleasure. He's the sunshine of the household and the comfort of our old age—and this reminds us of our text—"who would be without a baby?" Not we, if we can help it.

REMOVING TAN.—Tan can be removed from the face by dissolving magnesia in soft water; beat it to a thick mass, spread on the face, let it remain a few minutes, then wash off in castile soap suds, and rinse with soft water.

